# Lightweight Single Image Super-Resolution with Selective Channel Processing Network

**DOI:** 10.3390/s22155586

**Published:** 2022-07-26

**Authors:** Hongyu Zhu, Hao Tang, Yaocong Hu, Huanjie Tao, Chao Xie

**Affiliations:** 1 College of Mechanical and Electronic Engineering, Nanjing Forestry University, Nanjing 210037, China; hongyuzhu@njfu.edu.cn (H.Z.); thzypfy@163.com (H.T.); 2 School of Electrical Engineering, Anhui Polytechnic University, Wuhu 241000, China; yaoconghu@mail.ahpu.edu.cn; 3 School of Computer Science, Northwestern Polytechnical University, Xi’an 710072, China; huanjie_tao@nwpu.edu.cn; 4 College of Landscape Architecture, Nanjing Forestry University, Nanjing 210037, China

**Keywords:** single image super-resolution, lightweight image super-resolution, selective channel processing, differential channel attention

## Abstract

With the development of deep learning, considerable progress has been made in image restoration. Notably, many state-of-the-art single image super-resolution (SR) methods have been proposed. However, most of them contain many parameters, which leads to a significant amount of calculation consumption in the inference phase. To make current SR networks more lightweight and resource-friendly, we present a convolution neural network with the proposed selective channel processing strategy (SCPN). Specifically, the selective channel processing module (SCPM) is first designed to dynamically learn the significance of each channel in the feature map using a channel selection matrix in the training phase. Correspondingly, in the inference phase, only the essential channels indicated by the channel selection matrixes need to be further processed. By doing so, we can significantly reduce the parameters and the calculation consumption. Moreover, the differential channel attention (DCA) block is proposed, which takes into consideration the data distribution of the channels in feature maps to restore more high-frequency information. Extensive experiments are performed on the natural image super-resolution benchmarks (i.e., Set5, Set14, B100, Urban100, Manga109) and remote-sensing benchmarks (i.e., UCTest and RESISCTest), and our method achieves superior results to other state-of-the-art methods. Furthermore, our method keeps a slim size with fewer than 1 M parameters, which proves the superiority of our method. Owing to the proposed SCPM and DCA, our SCPN model achieves a better trade-off between calculation cost and performance in both general and remote-sensing SR applications, and our proposed method can be extended to other computer vision tasks for further research.

## 1. Introduction

Single image super-resolution (SR), aims at recovering a high-resolution image (HR) from its low-resolution image (LR) counterpart [1]. There is a recognized need for SR techniques in many fields [2,3,4,5,6,7], such as remote sensing, medical imaging, security surveillance, and hyperspectral images to name a few.

SR is a typically ill-posed problem because there is more than one solution for an LR input. Additionally, there is significant room for further improving the performance of SR. For these reasons, SR has been a subject of intense research for many years. Consequently, numerous SR methods have been proposed by researchers. These methods can be classified into three classes in general [8]: interpolation-based methods, reconstruction-based methods, and learning-based methods.

In the SR field, interpolation-based methods are usually the most straightforward and simple type, including the nearest interpolation, the bilinear interpolation, and the bicubic interpolation [9]. These methods tend to calculate the values of the unknown pixels using the known values of pixels surrounding them. Although these methods do not require large calculation consumption, they show poor ability to restore the edges, textures, and other high-frequency components of the pictures. The second category is the reconstruction-based methods [10], whose theoretical basis is the balanced and unbalanced sampling theorem. According to the degradation model, these methods reconstruct the high-resolution images by utilizing the complementarity of image information in the same scene. The reconstruction algorithms mainly include Maximum a Posteriori (MAP) [11,12], Projection onto Convex Sets (POCS) [13], and Iterative Back Projection (IBP) [14,15,16,17] methods. MAP regards the SR problem as a matter of statistical estimation. It has good performance in stability and denoise but shows poor ability in restoring the edges of pictures. IBP and POCS are all iterative algorithms, both of which have the disadvantage of multi-solution and a tremendous amount of calculation.

To progress in this area, researchers have proposed several machine learning (ML)-based SR methods [18,19]. The basic idea of these methods is to utilize the ML models to fit the relationship between the HR and LR images. The traditional ML-based methods include neighbor-embedding [20], sparse representation [8,21], and anchored neighborhood regression [22]. Although these methods achieved a performance improvement upon release, some problems still exist. First, a time-consuming process is needed to optimize the algorithms. Second, they have poor generalization ability when there is a gap in data distribution between the input and training datasets.

Since the pioneering work of SRCNN [23], which is proposed by Dong et al., deep-learning methods are becoming a common trend in SR research. In VDSR proposed by Kim et al. [24], the number of convolution layers increases to 20. In virtue of the residual learning strategy [25], the deepened network has a better performance compared to the shallow ones. Later, EDSR [26] was proposed, which is both a deep network with 60 layers and a wide one with 256 channels. Then, Zhang et al. further increased the network depth to over 100 and 400 layers in RDN [27] and RCAN [28], respectively. Compared with the approaches mentioned above, the deep-learning-based methods can achieve significantly higher performance under the criterion of peak signal-to-noise ratio (PSNR) and structure similarity (SSIM) [29]. However, the increase in layers and channels leads to an increase in computational consumption, which hinders these methods from being applied to real-world scenery.

In order to solve the problem of sizeable computational consumption, CARN-M [30] utilizes the recursive strategy to share the weight parameters of the blocks in the networks, which reduces the model size, simultaneously leading to a performance drop. Hui et al. [31] proposed the IDN model, which partially retained local short-path information. Furthermore, Hui et al. [32] proposed IMDN, which uses an information multi-distillation block to further improve the performance. Wang et al. [33] proposed SMSR, which explores sparsity in deep-learning SR models for efficient inference. To build a lightweight model with higher performance, we propose our convolution neural network with the selective channel processing strategy (SCPN). The SCPN model contains several selective channel processing modules (SCPM), which utilize the channel selection matrixes with learnable arguments to decide whether to process the channels in the feature map in the next convolution layer. Thus, it can save much calculation in the inference phase. Furthermore, for the sake of improving performance, we propose the differential channel attention block, which is modified from [28], and takes the distribution of channel parameters into consideration, which is more suitable for low-level vision tasks, and preserves more high-frequency textures and edges for SR reconstruction.

Overall, our contributions in this work can be summarized as follows:

(1) We propose the convolution neural network with selective channel processing strategy (SCPN). Extensive experiments prove that our model can achieve higher performance than previous works in remote sensing images in the SR field;
(2) We propose the selective channel processing module (SCPM), which contains trainable parameters in a channel selection matrix to decide whether to process the corresponding channels in the feature map in the next convolution layer. This strategy markedly reduces the calculation consumption and the model size;
(3) We propose the differential channel attention (DCA) block, which is more suitable for the SR tasks in restoring more high-frequency details and further improves the representation ability of the networks.

## 2. Related Work

SR is a classic low-level task in computer vision. We can coarsely divide the existing methods into two categories: the traditional methods and the deep-learning-based methods. Attention mechanism and adaptive inference are two typical strategies to improve the performance of the model. Due to space limitations, we offer a brief overview of the deep-learning SR methods, the attention mechanisms, and the adaptive inference strategy.

### 2.1. Deep-Learning SR Methods

With the rapid development of deep learning [34,35], recent years have witnessed a rapid rise in the growth of the deep-learning-based SR methods. As a pioneer work, Dong et al. [23] first proposed SRCNN. This method shows significant advantages over former methods, which uses a three-layer convolution neural network to learn the mapping function between LR and HR. In the year of 2016, Kim et al. [24] proposed the VDSR network, which is inspired by ResNet [25] architecture. Owing to the residual learning mechanism, the VDSR model deepens the network to 16 convolutional layers to learn more high-frequency prior knowledge. These methods mentioned above use interpolated images as input, resulting in additional computational waste. In order to effectively solve this problem, Shi et al. [36] proposed the ESPCN model, whose merit is the sub-pixel convolution layer for upscaling feature maps in the model. Due to the efficiency of this strategy, most of the following works use sub-pixel convolution layers in their models to promote performance enhancement and reduce calculation. To further tap the potential of CNN, Lim et al. [26] proposed a deep and wide network named EDSR, which won the 2017 NTIRE competition of SR. The previous works show that the width and depth of the network have a correlation with the performance in a certain range. However, for the lack of computing resources, the edge and mobile devices cannot support such large models; therefore, these methods lack practicality in real-world scenery.

### 2.2. Attention Mechanisms for SR

Zhang et al. [28] first proposed RCAN, which is the first attention model in the SR field. RCAN proposed the channel attention mechanism, which uses the global average pooling strategy to measure the importance of the features in each layer and calculates the weight of each channel by a multi-layer perceptron. In addition, Dai et al. [37] proposed SAN, which modified the channel attention using the covariance average pooling. RFANet, proposed by Liu et al. [38], takes advantage of the spatial attention mechanism to enhance the critical areas for better reconstructing features. Zhang et al. [39] proposed a non-local residual network for the image restoration task. In this work, they used the pixel-level non-local attention mechanism to capture long-distance spatial contextual information for SR reconstruction. Mei et al. [40] further explored the non-local attention at patch level with a cross-scale strategy. Recently, Liang et al. [41] proposed the SwinIR model, which is based on the epidemic Transformer [42,43] model to carry forward the self-attention mechanism in the SR field.

### 2.3. Adaptive Inference

Adaptive inference techniques have attracted increasing interest because of their ability to adapt the network structure based on the input. One typical type of adaptive inference is to select the path of inference at the levels of layers. Notably, Srivastava et al. [44] proposed the dropout strategy to prevent neural networks from overfitting, which performs as a pioneer in this field. Wu et al. [45] proposed the BlockDrop strategy, and implemented it on ResNet to drop several residual blocks to improve efficiency. Mullapudi et al. [46] proposed HydraNet, which has multiple branches and can dynamically choose a set for inferencing the results. Another type of adaptive inference technique is the early stopping strategy, which can skip at the location whenever it is judged to be unnecessary. Specifically, Figurnov et al. [47] proposed a spatially adaptive computation time strategy to terminate calculating at a space position where features are deemed good enough. Liu et al. [48] proposed the AdaSR model, which utilizes an adapter to adapt the number of convolutional layers implemented at different locations.

## 3. Visualization of Feature Maps in Classic Deep-Learning-Based SR Methods

In this section, we explore the sparsity of feature maps in the classic baseline networks, i.e., EDSR [26] and RCAN [28]. We choose butterfly.png in the Set5 [49] dataset as input, which is a typical case in the research of the SR field. We upscale the picture at the scale of 4 using the two models, and research the feature maps in detail.

**Figure 1:**
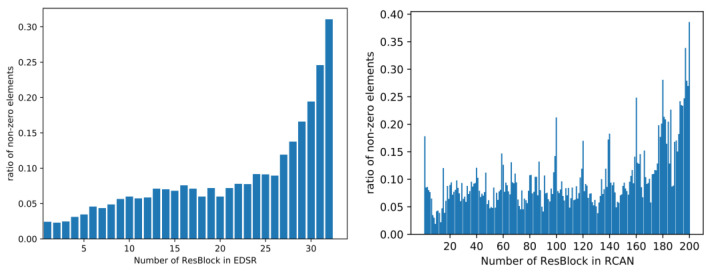
Ratio of the non-zero elements in the feature maps of EDSR and RCAN.

We investigate the proportion of the non-zero elements in the feature map after the ReLU layer in each block. Figure 1 illustrates a general trend where more elements are activated as the number of ResBlock grows. This means that the numerous elements in the feature maps in the front of the network are not as essential as those at the back of the network for reconstructing the final results.

**Figure 2:**
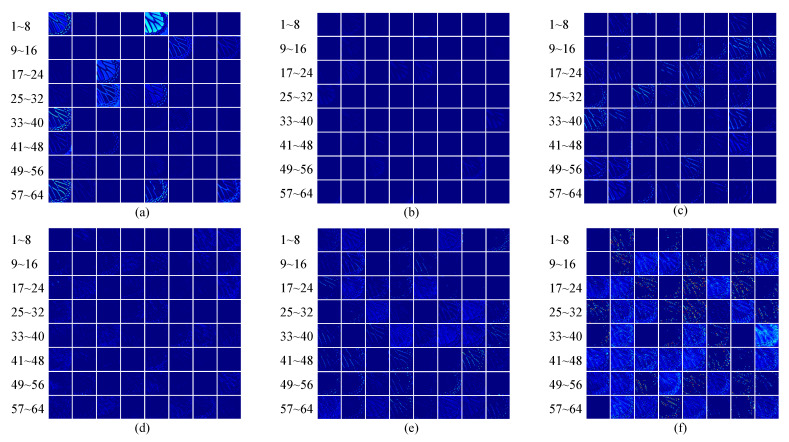
Visualization of the feature maps in RCAN. (**a**,**b**), respectively, represent the feature map in the first and last blocks in the first residual group. (**c**,**d**) represent the feature map in the first and last blocks in the fifth residual group. (**e**,**f**) represent the feature map in the first and last blocks in the last residual group.

Figure 2 shows the channels in the feature maps in the head, middle, and tail of the RCAN network. It is observed that many of the feature maps are filled with zeros, which contain little texture information. It is obvious that they contribute less to the process of reconstruction, which can be overlooked for simplicity and fast inference. In addition, Figure 2 demonstrates that the first and last feature maps of the backbone network store more activated textures than the ones in the middle of the network. Inspired by these observations, we propose the following selective channel processing network.

## 4. Selective Channel Processing Network (SCPN)

In this section, we first introduce the architecture of our proposed SCPN model. Then, we give a detailed description of the selective channel processing module and the differential channel attention block. Finally, we introduce the implementation details of the proposed SCPN.

### 4.1. Network Architecture

**Figure 3:**
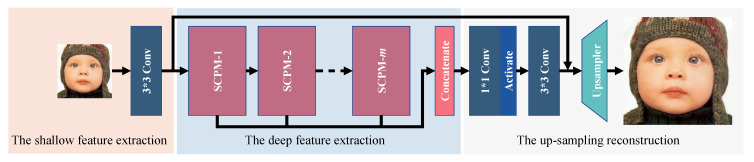
The whole architecture of our selective channel processing network (SCPN).

As shown in Figure 3, our selective channel processing network (SCPN) consists of three parts: the shallow feature extraction, the deep feature extraction, and the up-sampling reconstruction.

Given a low-resolution image $I_{LR}\;$ and its counterpart high-resolution image $I_{HR}$, the output of our model is denoted as $I_{SR}$. The shallow feature extraction consists of one convolution layer with the kernel size 3 × 3, following the earlier research [26,27,28,37,38,40], and the extracted features $F_{0}$ is represented as:

(1) $$F_{0}=P_{SFE}\left(I_{LR}\right),$$

where $P_{SFE}\left(\cdot \right)$ denotes the convolution operation. Then, $F_{0}$ is sent to the deep feature extraction part for extracting more effective features $F_{DF}$, which can be denoted as:

(2) $$F_{DF}=P_{DFE}\left(F_{0}\right),$$

where $P_{DFE}$ is the deep feature extraction part, which consists of *m* selective channel processing modules. Finally, we utilize the up-sampling reconstruction part to convert the deep features into output results, denoted as:

(3) $$I_{SR}=P_{UP}\left(F_{DF}+F_{0}\right),$$

where $P_{UP}$ means the up-sampling reconstruction, which contains the convolution layers and an up-sampler. Following [36], the up-sampler includes a convolution layer and a sub-pixel convolution, which also corresponds to our lightweight design principle.

**Figure 4:**
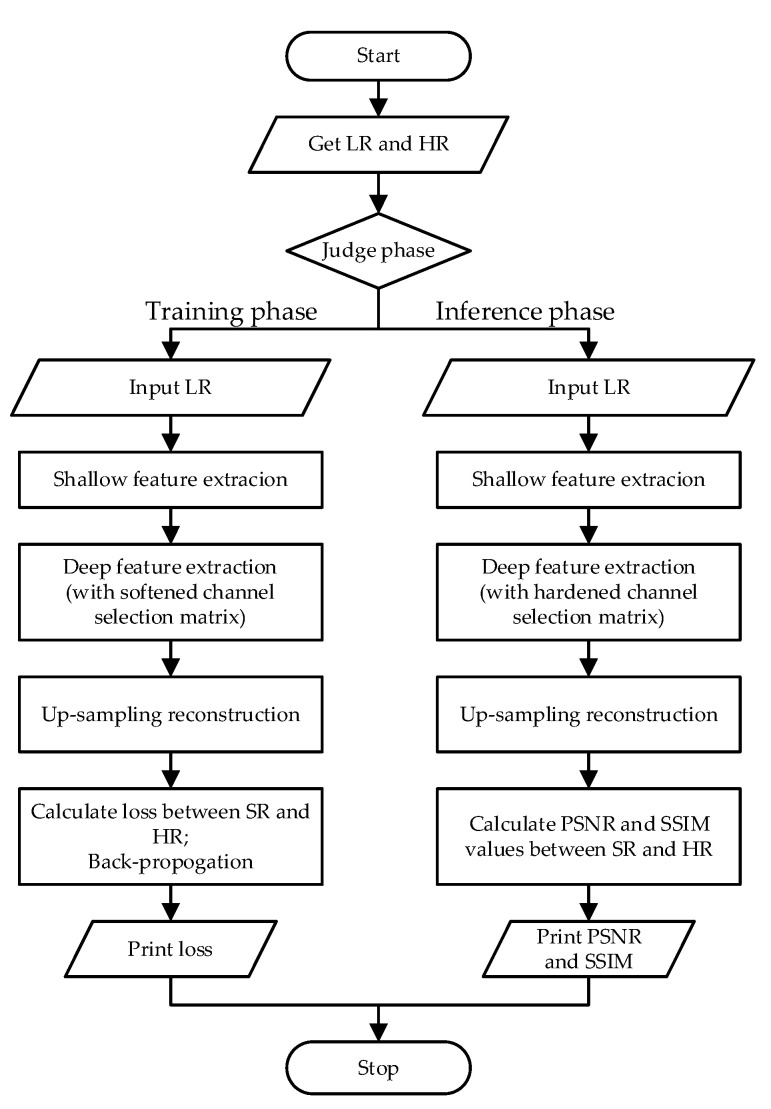
The flowchart of our proposed SCPN methodology.

To make the procedure of SCPN clearer, we present a flowchart in Figure 4.

### 4.2. Selective Channel Processing Module (SCPM)

**Figure 5:**
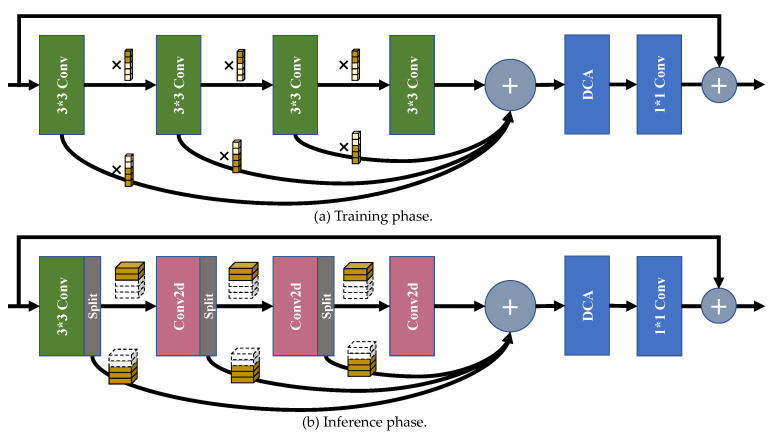
The sketch map of the selective channel processing module (SCPM). (**a**) is the flow path of feature maps in the training phase, while (**b**) is the flow path of feature maps in the inference phase. The combination of the small cubes in (**a**) denotes the channel selection matrix. The cuboids in the box denotes the feature maps generated by the previous convolution layer in (**b**). The activate layers are not shown for simplicity.

As shown in Figure 5, the proposed selective channel processing module (SCPM) has different forms in the training phase and the inference phase, which will be explicitly introduced below.

#### 4.2.1. SCPM in the Training Phase

**Channel selection matrix.** To set up the modules, channel selection matrixes are needed to learn to judge whether each channel in the feature maps is important or not in the feature maps generated by the convolution layers, and whether to transmit it to the next convolution layer. Ideally, we utilize the binary code, i.e., 0 and 1, to represent the ‘selection’ manipulations of the corresponding channels. To make the parameters of the channel selection matrix learnable, for the reason that the softmax function cannot convert the numbers close to the binary code, we adopt the Gumbel softmax distribution [50] to approximate the one-hot distribution. To be specific, for the *l*-th layer in the *m*-th SCPM, the channel selection matrix $CSM_{l}^{m}$ has two columns, and the number of rows in the matrix equals the number of channels. We input the parameters of the channel selection matrix into a Gumbel softmax function, and generate the parameters $M_{l}^{m}$ to reweight the feature maps output by the convolution layers:

(4) $$M_{l}^{m}\left[c,i\right]=\frac{\exp\left(\left(CSM_{l}^{m}\left[c,i\right]+G_{l}^{m}\left[c,i\right]\right)/\tau \right)}{\sum_{j=1}^{2}\exp\left(\left(CSM_{l}^{m}\left[c,j\right]+G_{l}^{m}\left[c,j\right]\right)/\tau \right)},$$

where *c* denotes the channel index, and $G_{l}^{m}\in {\mathbb{R}}^{C\times 2}$ represents the Gumbel noise tensor. In addition, *τ* denotes the temperature coefficient of the Gumbel softmax function. When *τ* tends to ∞, all results of Gumbel softmax function tends to 0.5, which makes the generated elements uniformly distributed. Conversely, when *τ* infinitely tends to 0, results from the function become one-hot, which makes the channel selection matrix binary fit our settings. When initializing the network architecture before the training phase, we use the random function to generate parameters for every SCM with Gaussian distribution $N\left(0,1\right)$. We denote the first column in $M_{l}^{m}$ as $M_{l,1}^{m}$, and the second column as $M_{l,2}^{m}$. 

**Architecture.** Figure 5a illustrates the flow path of the SCPM in the training phase. Four convolution layers are set for deeply processing the features from input. Let us denote the input feature map as $F_{in}$, the output of the *n*-th convolution layer as $F_{n}$, and the output feature map as $F_{out}$. Then we can get:

(5) $$F_{1}=Conv_{1}(F_{in}),$$

(6) $$F_{2}=Conv_{2}\left(M_{1,1}^{m}⊙F_{1}\right),$$

(7) $$F_{3}=Conv_{3}\left(M_{2,1}^{m}⊙F_{2}\right),$$

(8) $$F_{4}=Conv_{4}\left(M_{3,1}^{m}⊙F_{3}\right),$$

(9) $$F_{out}=Conv_{1\times 1}(DCA(M_{1,2}^{m}⊙F_{1}+M_{2,2}^{m}⊙F_{2}+M_{3,2}^{m}⊙F_{3}+F_{4}))+F_{in},$$

where $Conv_{n}$ denotes the *n*-th convolution layer, and ⊙ denotes the element-wise multiplication. DCA denotes the differential channel attention block, which will be detailed in the following article. 

**Training strategy.** During the training phase, we adjust the temperature coefficient *τ* with the following formula:

(10) $$\tau =\max\left(0.4,1-\frac{t}{500}\right),$$

where *t* is the number of epochs. It is shown that *τ* drops from 1 slightly to 0.4 at the 300 th epoch and maintains 0.4 during the following epochs in the training phase.

#### 4.2.2. SCPM in the Inference Phase

**Channel selection matrix.** Channel selection matrixes are properly optimized in the training phase in order to represent whether to preserve the channel to the next convolution layer, or directly send it to the addition layer at the end of the fourth convolution layer for feature-adding. In the inference phase, the channel selection matrixes work as a basis for the channel splitting processes. To get the binary code of the channel selection matrixes, for the two elements $M_{l}^{m}\left[c,1\right]$ and $M_{l}^{m}\left[c,2\right]$, we replace the larger one with 1 and the smaller one with 0 directly. In the channel selection matrix $CSM_{l}^{m}$, the positions of elements equal to 1 in the first column $M_{l,1}^{m}$ denote the coordinate number of channels to preserve to be sent to the next layer, and the positions of elements equal to 1 in the second column $M_{l,2}^{m}$ mean the coordinate number of channels to pass to the addition layer. 

**Architecture.** As shown in Figure 5b, the architecture of SCPM in the inference phase has a different shape from that in the training phase. The significant difference is that we introduce the channel splitting strategy to extract the channels from the output channels. For the *l*-th layer in the *m*-th SCPM, we first split the number of channels indicated by $M_{l,1}^{m}$ and then extract the convolution kernels at the corresponding positions in the next convolution layer in the meanwhile. Then, the two-dimensional convolutions are made using the extracted kernels and feature maps. 

To be explicit, the process of the inference phase can be denoted as:

(11) $$F_{1}=Conv_{1}\left(F_{in}\right),$$

(12) $$F_{2}=Conv2d\left(F_{1}\left[M_{1,1}^{m}\left(1\right)\right],w_{2}\left[M_{1,1}^{m}\left(1\right)\right]\right),$$

(13) $$F_{3}=Conv2d\left(F_{2}\left[M_{2,1}^{m}\left(1\right)\right],w_{3}\left[M_{2,1}^{m}\left(1\right)\right]\right),$$

(14) $$F_{4}=Conv2d\left(F_{3}\left[M_{3,1}^{m}\left(1\right)\right],w_{4}\left[M_{3,1}^{m}\left(1\right)\right]\right),$$

(15) $$F_{out}=Conv_{1\times 1}\left(DCA\left(F_{1}\left[M_{1,2}^{m}\left(1\right)\right]+F_{2}[M_{2,2}^{m}\left(1\right)\right]+F_{3}[M_{3,2}^{m}\left(1\right)\right]+F_{4}))+F_{in},$$

where $Conv_{1}$ means the first convolution layer, *Conv2d* means the 2-D convolution function, $F_{l}\left[M_{l,1}^{m}\left(1\right)\right]$ means the extracted feature map from the original feature map $F_{l}$, whose indexes equal to the positions of ‘1’ in $M_{l,1}^{m}$, and $w_{l}$ denotes the original weight of the *l*-th convolution layer. Other symbols have the same meanings as those in Section 4.2.1. With the combination of 2-D convolution and the selective channel processing strategy, we can avoid calculating the channels which contribute less for SR reconstruction in the feature maps, thus, we reduce the number of channels for calculating to a large extent, and do not have to store the parameters of the redundant convolutional kernels, therefore, save many redundant consumptions.

#### 4.2.3. Differential Channel Attention Block

**Figure 6:**
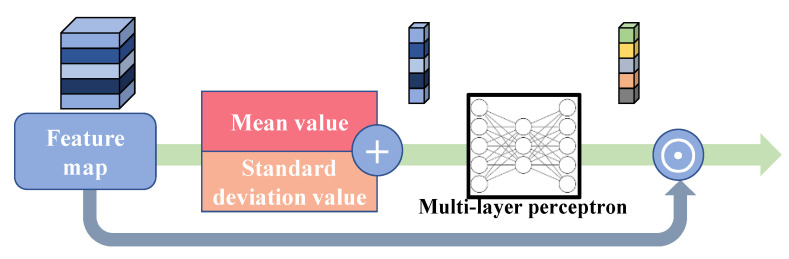
Differential channel attention block (DCA).

The channel attention mechanism is a widely used strategy in both high-level and low-level computer vision tasks. As a common practice, either global average pooling or global maximum pooling is utilized to generate the channel descriptor of the feature maps, and the channel descriptor will be processed to become the weight of each channel of the feature map. RCAN shows the advantage of this mechanism by achieving a higher rate of PSNR and SSIM. However, by only using the average value of each channel, we cannot extract the richer information from the feature map, e.g., the high-frequency details, the distribution and deviation of data, etc., therefore, having some negative impact on SR performance. To solve the problem and further boost the performance of our model, we propose the differential channel attention block (DCA), whose procedure is shown in Figure 6. We first calculate the mean value of each channel, whose formula is:

(16) $$mv_{c}=\frac{1}{H\times W}\underset{\left(i,j\right)\in x_{c}}{\sum }x_{c}\left[i,j\right],$$

where $mv_{c}$ means the mean value of the *c*-th channel, $x_{c}$ means the *c*-th channel of the input feature, (*i, j*) means the coordinate of the element in $x_{c}$, and *H* and *W* means the height and width of the channel feature, respectively. 

In the meanwhile, the standard deviation value of the input feature map is calculated, formulated as:

(17) $$sdv_{c}=\sqrt{\frac{1}{H\times W}\underset{\left(i,j\right)\in x_{c}}{\sum }{\left(x_{c}\left[i,j\right]-mv_{c}\right)}^{2}},\;$$

where $sdv_{c}$ means the standard deviation value of the *c*-th channel, and other symbols have the same meanings as in the formulas above. With the standard deviation value, we take the whole distribution of data into the model. Hence, our model has a better ability to reconstruct high-frequency information. This manipulation has the formula:

(18) sv=mv+sdv,

where *sv* means the summed value. Then, the addition operation is completed, and the summed values are sent to a multi-layer perceptron (MLP) for further processing. The MLP has three layers, where the first layer has 64 elements, the second 16, and the third 64. After this process, the weights of channels are formed. This process can be denoted as:

(19) $$y=MLP\left(sv\right),$$

where *y* denotes the generated weight of channels. Finally, we multiply the weights and the input feature, denoted as:

(20) output=x⊙y,

where ⊙ denotes the element-wise multiplication. With the plug-and-play DCA block, the proposed SCPN further upgrades its reconstruction performance.

#### 4.2.4. Implementation Details

As a supplement, we introduce the implementation details to explicitly explain our SCPN architecture. We set the number of the SCPMs as 6. There are four convolution layers in each SCPM whose kernel size is 3 × 3 and the zero-padding parameter is one and stride one. Another convolution layer in the SCPM has the kernel size of 1 × 1, the stride of 1 and no zero-paddings. The number of feature maps in our SCPN is set to 64 for better SR reconstruction results. In the up-sampling reconstruction section, the 3 × 3 convolution layer transforms the number of channels to $3\times r^{2}$, where *r* is the rate of SR. Then, the pixel-shuffle layer turns the number of channels to 3 (i.e., red, green, and blue channels), and the height and width of features become *r* times the original ones.

#### 4.2.5. Pseudocode of the Proposed Network

To better explain the procedure of our SCPN, we present the PyTorch-like pseudocode of the SCPN in the two phases (Algorithm 1).

**Algorithm 1** The PyTorch-like pseudocode of SCPN.
**###The basic module SCPM of SCPN.** def SCPM(input): if model.training:   c1=ReLU(conv1(input))  c1_0=c1*matrix1[0];c1_1=c1*matrix1[1]  c2=ReLU(conv2(c1_0))  c2_0=c2*matrix2[0];c2_1=c2*matrix2[1]  c3=ReLU(conv3(c2_0))  c3_0=c3*matrix3[0];c3_1=c3*matrix3[1]  c4=ReLU(conv4(c3))  c_out=c1_1+c2_1+c3_1+c4  out=conv5(CCA(c_out))+input  return out if model.inference:  pos1_0=position(matrix1[0]==1);pos1_1=position(matrix1[1]==1)  pos2_0=position(matrix2[0]==1);pos2_1=position(matrix2[1]==1)  pos3_0=position(matrix3[0]==1);pos3_1=position(matrix3[1]==1)  c1=F.conv2d(input,conv1.weight)  c1_0=split(c1,pos1_0);c1_1=split(c1,pos1_1)  c2=F.conv2d(c1_0,conv2.weight[pos1_0])  c2_0=split(c2,pos2_0);c2_1=split(c2,pos2_1)  c3=F.conv2d(c2_0,conv3.weight[pos2_0])  c3_0=split(c3,pos3_0);c3_1=split(c3,pos3_1)  c4=F.conv2d(c3_0,conv4.weight[pos3_0])  c_out=c1_1+c2_1+c3_1+c4  out=conv5(CCA(c_out))+input  return out **###The deep feature extraction part, which contains 6 SCPMs.** def P_DFE(input): out1=SCPM1(input) out2=SCPM2(out1) out3=SCPM3(out2) out4=SCPM4(out3) out5=SCPM5(out4) out6=SCPM6(out5) **###The whole SCPN model.** if model.training: F0=P_SFE(LR) #shallow feature extraction F1=P_DFE(F0) #deep feature extraction SR=P_UP(F0+F1) #upsampling reconstruction loss=sum(|HR-SR|)/(h*w) loss.backward() optimizer.step() if model.inference: F0=P_SFE(LR) F1= F1=P_DFE(F0) SR=P_UP(F0+F1) imshow(SR)

## 5. Experiments on General Images

### 5.1. Datasets and Evaluation Metrics

During the training phase, we utilize the DIV2K [51] dataset to construct our training set, which is widely used in the image restoration tasks, especially in the SR field. It contains 800 high-quality natural images with 2-K resolution and three channels of colors, i.e., red, green, and blue. For evaluating the performance of our SCPN, five standard benchmark datasets, i.e., Set5 [49], Set14 [52], B100 [53], Urban100 [54], and Manga109 [55] were selected as test sets. To be exact, Set5 and Set14 have 5 and 14 images without complex patterns, respectively. The B100 dataset contains 100 images of natural and cultural scenery. The Urban100 dataset comprises 100 images, whose semantics are about urban scenes. Manga109 contains 109 manga volumes drawn by professional manga artists in Japan. To build the low-resolution inputs in the datasets, we adopt the commonly used *imresize* function in MATLAB (www.mathworks.com, accessed on 23 July 2022), which utilizes the bicubic model for degradation.

In order to quantify the SR efficiency of our SCPN and its competitors, we adopt two universal standards, i.e., peak signal-to-noise ratio (PSNR) and structural similarity index (SSIM) [29] on the luminance channel in YCbCr space converted from RGB space. In simple terms, PSNR calculates the pixel-wise differences between the super-resolved images and the ground truth. At the same time, SSIM indicates the structural similarity, e.g., luminance, contrast, and structures between the two images. The higher scores of the evaluation metrics mean the better performance of the model.

### 5.2. Training Details

A pretreatment was carried out before training. That is, we subtracted the mean value from images in the training set. During the training phase, we crop the low-resolution images to patches whose height and width fit 192/*r*, where *r* is the rate of SR upscaling. Corresponding high-resolution images are cropped in the meanwhile to be the labels for training. Data augmentation was conducted after the data loader read the images, that is, random 90° rotations and horizontal flips. We trained our model with the L1 loss function and ADAM optimizer [56], whose hyper-parameters are: *β*_1_ = 0.9, *β*_2_ = 0.999, and *ϵ* = 10^−8^. The initial learning rate was set to 2 × 10^−4^, then decreased to half after every 400 epochs for SR upscale rates of 2 and 3, and after every 500 epochs for an SR upscale rate of 4. The minibatch size was set to 16. We implemented all the experiments using the PyTorch framework on a workstation with an NVIDIA (www.nvidia.com, accessed on 23 July 2022) RTX2080Ti GPU.

### 5.3. Effectiveness of Selective Channel Processing Strategy

To demonstrate the effect of our proposed selective channel processing strategy, we designed two new variant modules, i.e., Module-A and Module-B, to replace the original SCPM in our SCPN, and trained them with the same strategy.

**Figure 7:**
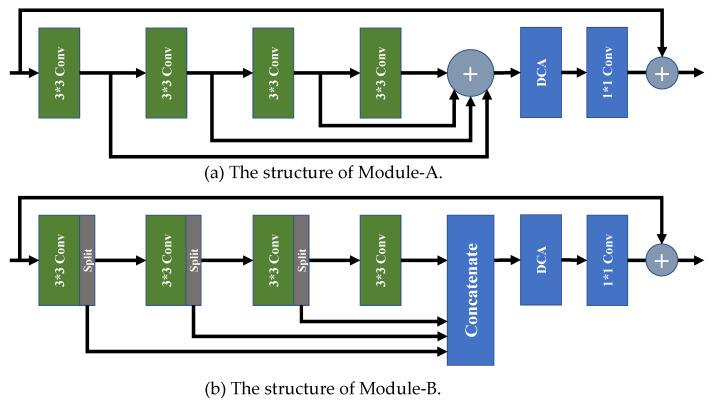
The structure of Module-A and Module-B.

As shown in Figure 7a, all the feature maps generated by the convolution layers are added by the addition layer without cooperating with the channel selection matrixes. In Figure 7b, for the feature maps with channel numbers of 64, 16 channels in front are split to pass to the concatenating layer, and the rest are preserved to be sent into the next convolution layer for further processing. As a comparison, our SCPM selects which channels to preserve or pass to the addition layer to skip processing with a learnable channel selection matrix. Comparative results are shown in Table 1.

As illustrated in Table 1, the network with Module-A, which has no channel selection matrix to selectively pass the channels to the next layer, shows a significant performance drop. The main reason is that redundant features are passed to the following convolution layer in the Module-A architecture, and this degrades the SR performance. In our SCPN, the channel selection matrix can pass the feature pieces, which are needed by the next convolution layer, and pass the rest of the channels to the addition layer, which leads to fewer parameters, less computational cost, and higher performance of PSNR and SSIM. An inspection of the table shows that the network with Module-B has fewer parameters than our SCPN. Although the architecture of Module-B seems more lightweight, its strategy is only to pass a fixed quantity of channels to the next convolution layer, and aggregate the rest channels in the concatenation layer, which leads to the lack of processing a proportion of features for SR reconstruction. Owing to our selective channel processing strategy, our SCPN achieves a better trade-off between computational complexity and performance.

### 5.4. Visualization of Channel Selection Matrixes

**Figure 8:**
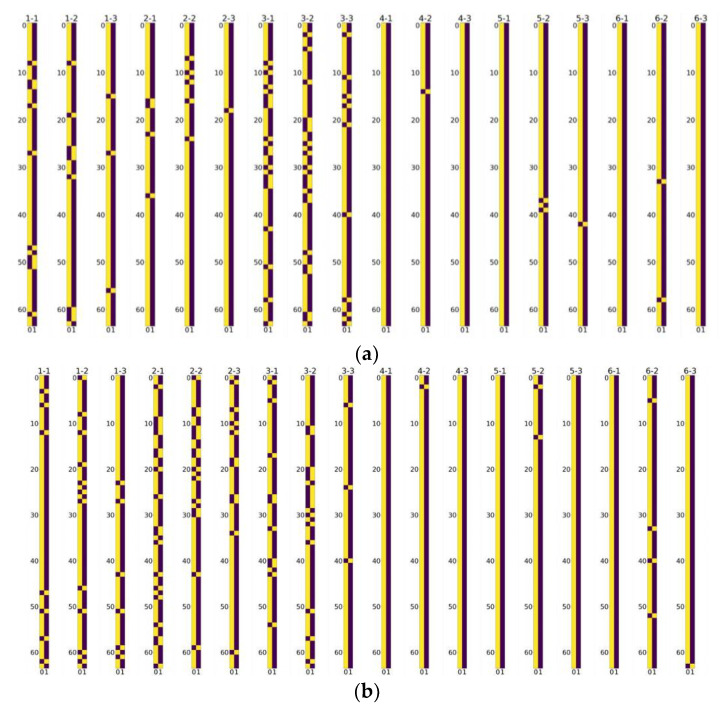

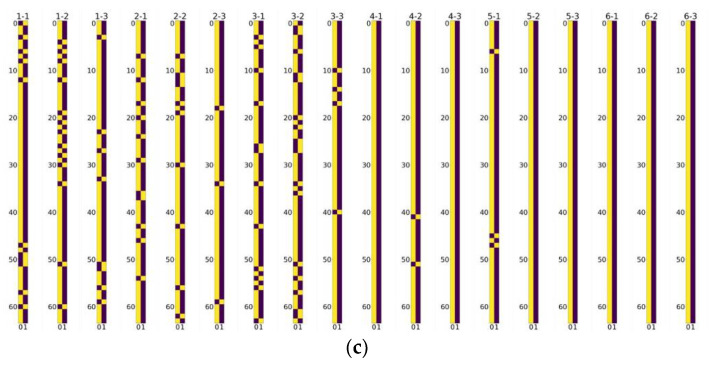
Visualization of the selective channel matrixes, where yellow denotes 1 and brown denotes 0 in the figures. *X*-*Y* denotes the SCM for the *Y*-th layer in the *X*-th SCPM. (**a**) Channel selection matrixes in SCPN of upscale factor 2. (**b**) Channel selection matrixes in SCPN of upscale factor 3. (**c**) Channel selection matrices in SCPN of upscale factor 4.

We visualize the selective channel matrixes in Figure 8. It is observed that, in models of any scale factor, more channels in the feature map are preserved in the layers in the tail of the model than those in the front. This illustrates that more features in the front have less significance, which can be stridden over to avoid computational redundancy. Figure 7 also shows that more layers are preserved to be sent into the next layers than the ones sent to the addition layer, which demonstrates that most of the channels in the feature maps are of significance for reconstructing the final SR results. These observations also echo the phenomena shown in Section 3.

### 5.5. Quantitative Evaluation and Visual Comparision

In order to test the effectiveness of the proposed model, we compare the SCPN with the bicubic interpolation method and nine state-of-the-art models, including SRCNN [23], FSRCNN [57], VDSR [24], DRCN [58], LapSRN [59], SRFBN-S [60], CARN [30], IDN [31], and IMDN [32]. Since we mainly focus on the lightweight network designs in this paper, several recent works with more than 2 M parameters (e.g., EDSR [26] (~40 M), RCAN [28] (~15 M), and SAN [37] (~15 M)) are not included for comparison. We report the quantitative comparison in Table 2.

#### 5.5.1. Quantitative Results

It can be seen from Table 2, that our SCPN outperforms the state-of-the-art methods with a higher PSNR and SSIM value. Our method also keeps a slim model size, which holds its parameters within one million.

Explicitly, the bicubic interpolation method has no prior knowledge for the SR reconstruction, therefore, it has inferior performance. SRCNN modifies the interpolated images with a shallow network architecture, which achieves a 2~3 dB progress in PSNR over the interpolation methods. FSRCNN and VDSR further increase the number of layers but do not achieve rapid growth due to the limitation of the network architectures. DRCN and SRFBN-S utilize the recursive mechanism, which can recurrently use the modules in the networks with the shared parameters. This mechanism saves the parameters but limits the network to learn more prior knowledge. IDN and IMDN propose and enhance the information distillation mechanism, respectively, which helps to reconstruct SR images without too many parameters. Our proposed SCPN utilizes the selective channel processing strategy, which empowers the network to save parameters and achieve better performance. Our proposed method surpasses all the methods above and achieves state-of-the-art performance and keeps a slim model size in the meanwhile.

#### 5.5.2. Qualitative Results

**Figure 9:**
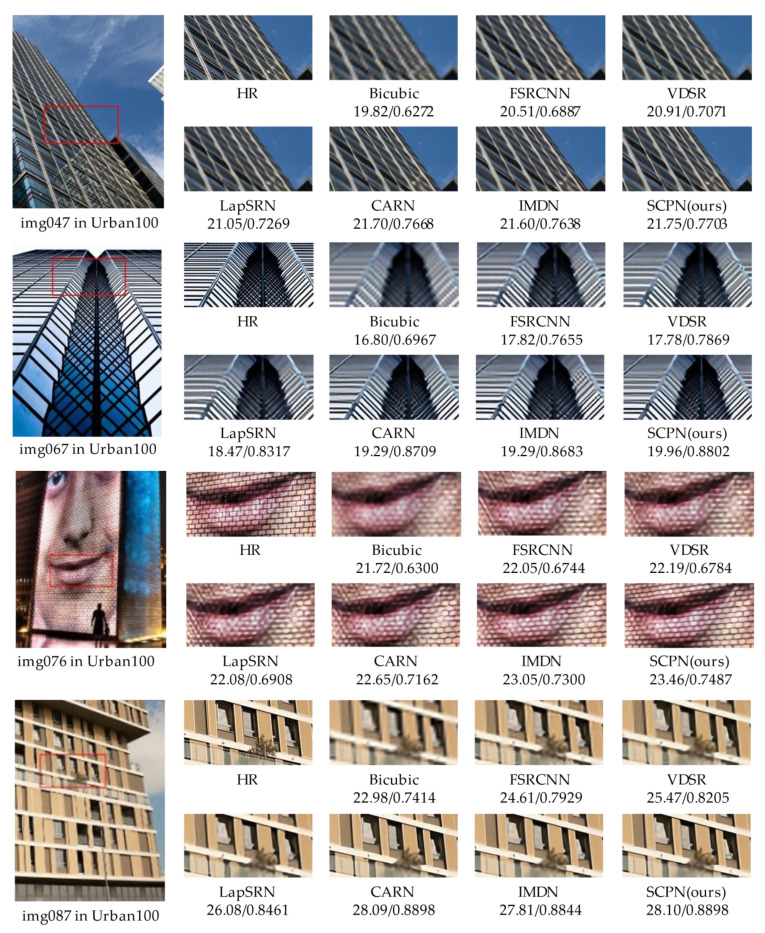
Qualitative results for ×4 SR. The numbers under the name of methods means the PSNR and SSIM values of the corresponding results.

We provide the visual comparison of some selected pictures (i.e., img047, img067, img076, and img087 in the Urban100 dataset) generated by our SCPN and other previous works, which are shown in Figure 9. First, take img047 and img087 as an example. The difficulty in reconstructing the images is to show the edges of the windows in the buildings. Our SCPN can precisely recover the edges, making the SR images look sharper than others. Regarding img067, our SCPN performs better when facing complex textures in comparison with the other methods. It should be noted that our method recognizes the two-line stripes and tries to make them clearer, while other methods ignore this detail. In img076, our SCPN restores the blocks on the wall with more regular textures, and other methods cannot reconstruct these rectangles. In sum, our proposed SCPN model generates clearer SR results than other methods, especially in detailed sections. Owing to the selective channel processing strategy and DCA, our method achieves the best performance with limited parameters.

## 6. Remote Sensing Image Super-Resolution

Remote sensing technology is now widely used in agriculture, forestry, military, and other fields. As enhancing the quality of remote sensing images is of great significance, we conducted experiments on remote sensing datasets in order to fit our method with the remote sensing field. Because of the difference of shooting angels and the existing distribution bias between the natural and remote sensing images, we utilized the pre-trained model with the DIV2K dataset and fine-tuned it on the remote sensing dataset. Owing to transferring the external knowledge from the natural image domain to the remote sensing image domain, our model achieves faster convergence and better performance in the remote sensing SR tasks.

We conducted experiments on the UC Merced Land-use [61] dataset, which is used by most remote sensing SR methods. The UC Merced Land-use dataset is one of the most famous datasets in the remote sensing research area. It contains 21 classes of land-use scenes, and each class includes 100 aerial images with a high spatial resolution (i.e., 0.3 m/pixel) and size of 256 × 256. Following the settings of the previous works [7,62], we randomly selected 40 images per class (i.e., totally 840 images) to construct the training set, and randomly chose 40 images in the training set as a validation set. Furthermore, we constructed the UCTest dataset with the 120 randomly selected images from the remaining part of the dataset. The acquisition of the HR-LR pairs for training and testing is the same as that for the common images in Section 5.1. The training strategy for remote sensing images is the same as that for common images in Section 5.2, and the only difference is that we load the weight of the model trained by common datasets for the transfer strategy mentioned above. We also trained the IMDN model with the same strategy for comparison.

The NWPU-RESISC45 dataset [63] is a publicly available benchmark dataset, which covers 45 classes with 700 images in each class extracted from Google Earth. We randomly chose 180 images from NWPU-RESISC45 to make up a test dataset named RESISCTest to evaluate the performance and generalization ability of our model.

Table 3 shows the mean PSNR and SSIM value of test datasets by the compared methods. We can observe that our SCPN achieves a higher PSNR value (approximately 0.1 dB), and a higher SSIM index (approximately 0.004) than its main competitor, i.e., IMDN. It is noteworthy that IMDN-T achieved its best performance after more than 1000 epochs of fine-tuning, while our SCPN only needs 8 epochs of fine-tuning to achieve its best performance, which illustrates that our method has better generalization ability and is easier for training.

**Figure 10:**
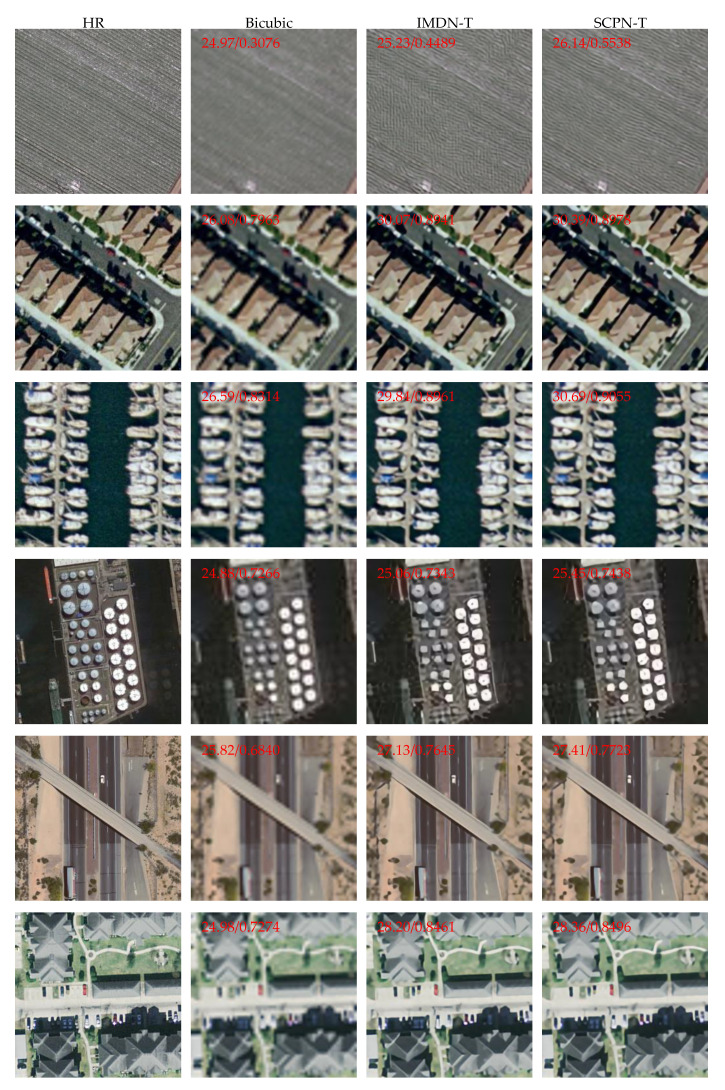
Visual results for remote-sensing images. The numbers in the upper left corner of the images denotes the PSNR and SSIM values of themselves.

To fully demonstrate the effectiveness of our method, we provide six visual results of the scale factor ×4 in the two test datasets, which are shown in Figure 10. The results shown illustrate that our SCPN-T restores more high-frequency information precisely and reconstructs remote sensing pictures with better visual effects.

**Figure 11:**
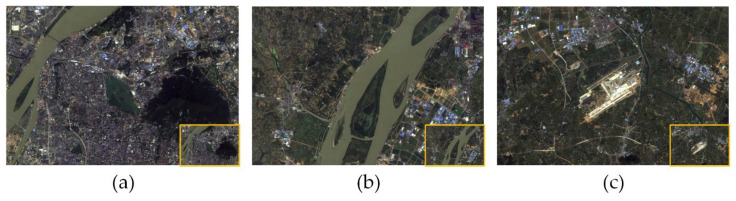
The visual results of the landscapes around (**a**) Xuanwu Lake, (**b**) Xinjizhou National Wetland Park, and (**c**) Lukou International Airport in Nanjing from the Landsat-8 dataset, with upscale factor 4 generated by our method. The small pictures in the right button corner are the low-resolution images, and the larger ones are high-resolution images.

**Application in real-world cases**. To further test the performance of our method in real-world scenes, we captured three remote-sensing images from the Landsat-8 satellite [64,65,66,67], which are the landscapes around Xuanwu Lake, Xinjizhou National Wetland Park, and Lukou International Airport in Nanjing. The original size of these images is 900 × 619. Our method successfully super-resolved these images with good visual effects and abundant details, which are shown in Figure 11. It is demonstrated that our proposed method can be well-applied to real-world remote-sensing scenery.

## 7. Conclusions

In this paper, we propose a lightweight convolution neural network with the selective channel processing strategy (SCPN) for single image super-resolution. Specifically, we propose selective channel processing modules (SCPM) to execute our selective channel processing strategy, which utilizes channel selection matrixes with learnable parameters. In the training phase, selective channel matrixes are softened and multiple the corresponding feature maps to guide the model distinguish the importance of each channel. In the inference phase, the values in the selective channel matrixes are hardened to work as the gates, which decide whether to process the corresponding channels in the next convolution layer or pass the channels to the addition layer directly for simplified calculation. What is more, we propose the differential channel attention block in order to restore more high-frequency details. Extensive experiments demonstrate that our method achieves a better trade-off between model complexity and performance, which keeps the number of parameters within 1 M, and gets higher PSNR and SSIM values of the test datasets beyond its competitors. Section 5 and Section 6 show that our method can generate natural images and remote-sensing images with higher quality and fine details and achieve better results beyond previous state-of-the-art methods both in quantitative and qualitative comparisons. Specifically, our SCPN achieves an approximately 0.1 dB higher PSNR value and 0.004 higher SSIM value beyond IMDN, its main competitor. In the future, we will explore efficient ways to deploy our lightweight model on mobile devices. At the same time, we will explore the other lightweight strategies in the SR field, such as introducing the sparsity convolution in the models to further reduce the size and calculation complexity of our models.

## Figures and Tables

**Table 1:** The comparison of complexity and performance on Set14 by the contestants with upscale factor 4. FLOPs denote the number of floating-point operations.

Model	Parameters	FLOPs	PSNR
Network with Module-A	1.01 M	115.5 G	28.56
Network with Module-B	0.72 M	82.0 G	28.56
SCPN	0.95 M	115.3 G	28.60

**Table 2:** Quantitative results of the compared methods in the format of PSNR/SSIM. #Params denotes parameters for short. The results are either reproduced by ourselves with the official settings or copied directly from the origin paper. Bold numbers indicate the best performance.

Method	Scale	#Params	Set5	Set14	B100	Urban100	Manga109
Bicubic [9]	×2	-	33.66/0.9299	30.24/0.8688	29.56/0.8431	26.88/0.8403	30.80/0.9339
SRCNN [23]		8 K	36.66/0.9542	32.45/0.9067	31.36/0.8879	29.50/0.8946	35.60/0.9663
FSRCNN [57]		13 K	37.05 0.9560	32.66 0.9090	31.53 0.8920	29.88 0.9020	36.67 0.9710
VDSR [24]		665 K	37.53/0.9590	33.05/0.9130	31.90/0.8960	30.77/0.9140	37.22/0.9750
DRCN [58]		1774 K	37.63/0.9588	33.04/0.9118	31.85/0.8942	30.75/0.9133	37.55/0.9732
LapSRN [59]		813 K	37.52/0.9591	33.08/0.9130	31.08/0.8950	30.41/0.9101	37.27/0.9740
SRFBN-S [60]		282 K	37.78/0.9597	33.35/0.9156	32.00/0.8970	31.41/0.9207	38.06/0.9757
CARN [30]		1592 K	37.76/0.9590	33.52/0.9166	32.09/0.8978	31.92/0.9256	38.36/0.9765
IDN [31]		553 K	37.83/0.9600	33.30/0.9148	32.08/0.8985	31.27/0.9196	38.01/0.9749
IMDN [32]		694 K	38.00/0.9605	33.54/0.9172	32.16/0.8994	32.09/0.9279	38.73/0.9771
SCPN (ours)		938 K	**38.08/0.9607**	**33.65/0.9177**	**32.19/0.89967**	**32.23/0.9288**	**38.89/0.9774**
Bicubic [9]	×3	-	30.39/0.8682	27.55/0.7742	27.21/0.7385	24.46/0.7349	26.95/0.8556
SRCNN [23]		8 K	32.75/0.9090	29.30/0.8215	28.41/0.7863	26.24/0.7989	30.48/0.9117
FSRCNN [57]		13 K	33.18/0.9140	29.37/0.8240	28.53/0.7910	26.43/0.8080	31.10/0.9210
VDSR [24]		665 K	33.66/0.9213	29.77/0.8314	28.82/0.7976	27.14/0.8279	32.01/0.9340
DRCN [58]		1774 K	33.82/0.9226	29.76/0.8311	28.80/0.7963	27.15/0.8276	32.24/0.9343
LapSRN [59]		502 K	33.81/0.9220	29.79/0.8325	28.82/0.7980	27.07/0.8275	32.21/0.9350
SRFBN-S [60]		375 K	34.20/0.9255	30.10/0.8372	28.96/0.8010	27.66/0.8415	33.02/0.9404
CARN [30]		1592 K	34.29/0.9255	30.29/0.8407	29.06/0.8034	28.06/0.8493	33.50/0.9440
IDN [31]		553 K	34.11/0.9253	29.99/0.8354	28.95/0.8013	27.42/0.8359	32.71/0.9381
IMDN [32]		703 K	34.42/0.9275	30.25/0.8401	29.06/0.8041	28.12/0.8507	33.49/0.9440
SCPN (ours)		934 K	**34.44/0.9275**	**30.33/0.8420**	**29.09/0.8046**	**28.18/0.8522**	**33.62/0.9445**
Bicubic [9]	×4	-	28.42/0.8104	26.00/0.7027	25.96/0.6675	23.14/0.6577	24.89/0.7866
SRCNN [23]		8 K	30.48/0.8628	27.50/0.7513	26.90/0.7101	24.52/0.7221	27.58/0.8555
FSRCNN [57]		13 K	30.72/0.8660	27.61/0.7550	26.98/0.7150	24.62/0.7280	27.90/0.8610
VDSR [24]		665 K	31.35/0.8838	28.01/0.7674	27.29/0.7251	25.18/0.7524	28.83/0.8870
DRCN [58]		1774 K	31.53/0.8854	28.02/0.7670	27.23/0.7233	25.14/0.7510	28.93/0.8854
LapSRN [59]		502 K	31.54/0.8850	28.19/0.7720	27.32/0.7270	25.21/0.7560	29.09/0.8900
SRFBN-S [60]		483 K	31.98/0.8923	28.45/0.7779	27.44/0.7313	25.71/0.7719	29.91/0.9008
CARN [30]		1592 K	32.13/0.8937	28.60/0.7806	27.58/0.7349	26.07/0.7837	30.47/0.9084
IDN [31]		553 K	31.82/0.8903	28.25/0.7730	27.41/0.7297	25.41/0.7632	29.41/0.8942
IMDN [32]		715 K	32.19/0.8943	28.56/0.7807	27.53/0.7345	26.02/0.7825	30.32/0.9057
SCPN (ours)		952 K	**32.20/0.8948**	**28.60/0.7819**	**27.55/0.7354**	**26.10/0.7857**	**30.49/0.9080**

**Table 3:** Quantitative results of the compared methods for scale factor ×4. Bold numbers indicate the best performance. The suffix T denotes that the parameters in the model are after tuning.

Method	UCTest		RESISCTest	
	PSNR	SSIM	PSNR	SSIM
Bicubic	26.77	0.6968	26.43	0.6300
IMDN-T	29.19	0.7920	26.44	0.6369
SCPN-T	29.32	0.7961	26.53	0.6406

## Data Availability

The training data presented in the study are openly available at https://cv.snu.ac.kr/research/EDSR/DIV2K.tar, accessed in 2017.

## References

[1] Anwar S., Khan S., Barnes N. (2020). A Deep Journey into Super-resolution: A Survey. ACM Comput. Surv..

[2] Xie C., Zeng W.L., Lu X.B. (2019). Fast Single-Image Super-Resolution via Deep Network With Component Learning. IEEE Trans. Circuits Syst. Video Technol..

[3] Dong X., Xi Z., Sun X., Gao L. (2019). Transferred Multi-Perception Attention Networks for Remote Sensing Image Super-Resolution. Remote Sens..

[4] Gu J., Sun X., Zhang Y., Fu K., Wang L. (2019). Deep Residual Squeeze and Excitation Network for Remote Sensing Image Super-Resolution. Remote Sens..

[5] Li L., Zhang S., Jiao L., Liu F., Yang S., Tang X. (2019). Semi-Coupled Convolutional Sparse Learning for Image Super-Resolution. Remote Sens..

[6] Li X., Zhang L., You J. (2019). Domain Transfer Learning for Hyperspectral Image Super-Resolution. Remote Sens..

[7] Wang Y., Zhao L., Liu L., Hu H., Tao W. (2021). URNet: A U-Shaped Residual Network for Lightweight Image Super-Resolution. Remote Sens..

[8] Xie C., Zeng W.L., Jiang S.Q., Lu X.B. (2017). Multiscale self-similarity and sparse representation based single image super-resolution. Neurocomputing.

[9] Keys R. (1981). Cubic convolution interpolation for digital image processing. IEEE Trans. Acoust. Speech Signal Process..

[10] Lin Z.C., Shum H.Y. (2004). Fundamental limits of reconstruction-based superresolution algorithms under local translation. IEEE Trans. Pattern Anal. Mach. Intell..

[11] Freeman W.T., Jones T.R., Pasztor E.C. (2002). Example-based super-resolution. IEEE Comput. Graph. Appl..

[12] Freeman W.T., Pasztor E.C., Carmichael O.T. (2000). Learning low-level vision. Int. J. Comput. Vis..

[13] Sung Cheol P., Min Kyu P., Moon Gi K. (2003). Super-resolution image reconstruction: A technical overview. IEEE Signal Process. Mag..

[14] Irani M., Peleg S. Super Resolution from Image Sequences. Proceedings of the 1990 10th International Conference on Pattern Recognition.

[15] Irani M., Peleg S. (1991). Improving resolution by image registration. CVGIP Graph. Models Image Process..

[16] Irani M., Peleg S. (1991). Image Sequence Enhancement Using Multiple Motions Analysis.

[17] Irani M., Peleg S. (1993). Motion analysis for image enhancement: Resolution, occlusion, and transparency. J. Vis. Commun. Image Represent..

[18] Yan X.A., Liu Y., Xu Y.D., Jia M.P. (2020). Multistep forecasting for diurnal wind speed based on hybrid deep learning model with improved singular spectrum decomposition. Energy Convers. Manag..

[19] Yan X.A., Liu Y., Xu Y.D., Jia M.P. (2021). Multichannel fault diagnosis of wind turbine driving system using multivariate singular spectrum decomposition and improved Kolmogorov complexity. Renew. Energy.

[20] Chang H., Yeung D.-Y., Xiong Y. Super-Resolution through Neighbor Embedding. Proceedings of the 2004 IEEE Computer Society Conference on Computer Vision and Pattern Recognition, CVPR 2004.

[21] Xie C., Zeng W.L., Jiang S.Q., Lu X.B. (2018). Bidirectionally Aligned Sparse Representation for Single Image Super-Resolution. Multimed. Tools Appl..

[22] Timofte R., Smet V.D., Gool L.J.V. A+: Adjusted Anchored Neighborhood Regression for Fast Super-Resolution. Proceedings of the Asian Conference on Computer Vision.

[23] Dong C., Loy C.C., He K., Tang X. (2016). Image Super-Resolution Using Deep Convolutional Networks. IEEE Trans. Pattern Anal. Mach. Intell..

[24] Kim J., Lee J., Lee K.M. Accurate Image Super-Resolution Using Very Deep Convolutional Networks. Proceedings of the 2016 IEEE Conference on Computer Vision and Pattern Recognition (CVPR).

[25] He K., Zhang X., Ren S., Sun J. Deep Residual Learning for Image Recognition. Proceedings of the IEEE Conference on Computer Vision and Pattern Recognition.

[26] Lim B., Son S., Kim H., Nah S., Mu Lee K. Enhanced Deep Residual Networks for Single Image Super-Resolution. Proceedings of the IEEE Conference on Computer Vision and Pattern Recognition Workshops.

[27] Zhang Y., Tian Y., Kong Y., Zhong B., Fu Y. Residual Dense Network for Image Super-Resolution. Proceedings of the 2018 IEEE/CVF Conference on Computer Vision and Pattern Recognition.

[28] Zhang Y., Li K., Li K., Wang L., Zhong B., Fu Y. Image Super-Resolution Using Very Deep Residual Channel Attention Networks. Proceedings of the European Conference on Computer Vision ECCV.

[29] Zhou W., Bovik A.C., Sheikh H.R., Simoncelli E.P. (2004). Image quality assessment: From error visibility to structural similarity. IEEE Trans. Image Process..

[30] Ahn N., Kang B., Sohn K.A. Fast, Accurate, and Lightweight Super-Resolution with Cascading Residual Network. Proceedings of the 15th European Conference on Computer Vision (ECCV).

[31] Hui Z., Wang X.M., Gao X.B. Fast and Accurate Single Image Super-Resolution via Information Distillation Network. Proceedings of the 31st IEEE/CVF Conference on Computer Vision and Pattern Recognition (CVPR).

[32] Hui Z., Gao X.B., Yang Y.C., Wang X.M. Lightweight Image Super-Resolution with Information Multi-distillation Network. Proceedings of the 27th ACM International Conference on Multimedia (MM).

[33] Wang L.G., Dong X.Y., Wang Y.Q., Ying X.Y., Lin Z.P., An W., Guo Y.L. Exploring Sparsity in Image Super-Resolution for Efficient Inference. Proceedings of the IEEE/CVF Conference on Computer Vision and Pattern Recognition (CVPR), Electr Network.

[34] Si W., Xiong J., Huang Y.P., Jiang X.S., Hu D. (2022). Quality Assessment of Fruits and Vegetables Based on Spatially Resolved Spectroscopy: A Review. Foods.

[35] Yan X.A., Liu Y., Jia M.P. (2020). Multiscale cascading deep belief network for fault identification of rotating machinery under various working conditions. Knowl.-Based Syst..

[36] Shi W., Caballero J., Huszár F., Totz J., Aitken A.P., Bishop R., Rueckert D., Wang Z. Real-Time Single Image and Video Super-Resolution Using an Efficient Sub-Pixel Convolutional Neural Network. Proceedings of the 2016 IEEE Conference on Computer Vision and Pattern Recognition (CVPR).

[37] Dai T., Cai J., Zhang Y.-B., Xia S., Zhang L. Second-Order Attention Network for Single Image Super-Resolution. Proceedings of the 2019 IEEE/CVF Conference on Computer Vision and Pattern Recognition (CVPR).

[38] Liu J., Zhang W., Tang Y., Tang J., Wu G. Residual feature aggregation network for image super-resolution. Proceedings of the IEEE/CVF Conference on Computer Vision and Pattern Recognition.

[39] Zhang Y., Li K., Li K., Zhong B., Fu Y. Residual Non-local Attention Networks for Image Restoration. Proceedings of the International Conference on Learning Representations.

[40] Mei Y., Fan Y., Zhou Y., Huang L., Huang T., Shi H. Image Super-Resolution With Cross-Scale Non-Local Attention and Exhaustive Self-Exemplars Mining. Proceedings of the 2020 IEEE/CVF Conference on Computer Vision and Pattern Recognition (CVPR).

[41] Liang J.Y., Cao J.Z., Sun G.L., Zhang K., Van Gool L., Timofte R., Soc I.C. SwinIR: Image Restoration Using Swin Transformer. Proceedings of the IEEE/CVF International Conference on Computer Vision (ICCVW).

[42] Vaswani A., Shazeer N., Parmar N., Uszkoreit J., Jones L., Gomez A.N., Kaiser L., Polosukhin I. Attention Is All You Need. Proceedings of the 31st Annual Conference on Neural Information Processing Systems (NIPS).

[43] Dosovitskiy A., Beyer L., Kolesnikov A., Weissenborn D., Zhai X., Unterthiner T., Dehghani M., Minderer M., Heigold G., Gelly S. (2020). An image is worth 16×16 words: Transformers for image recognition at scale. arXiv.

[44] Srivastava N., Hinton G., Krizhevsky A., Sutskever I., Salakhutdinov R. (2014). Dropout: A Simple Way to Prevent Neural Networks from Overfitting. J. Mach. Learn. Res..

[45] Wu Z.X., Nagarajan T., Kumar A., Rennie S., Davis L.S., Grauman K., Feris R. BlockDrop: Dynamic Inference Paths in Residual Networks. Proceedings of the 31st IEEE/CVF Conference on Computer Vision and Pattern Recognition (CVPR).

[46] Mullapudi R.T., Mark W.R., Shazeer N., Fatahalian K. HydraNets: Specialized Dynamic Architectures for Efficient Inference. Proceedings of the 31st IEEE/CVF Conference on Computer Vision and Pattern Recognition (CVPR).

[47] Figurnov M., Collins M.D., Zhu Y.K., Zhang L., Huang J., Vetrov D., Salakhutdinov R. Spatially Adaptive Computation Time for Residual Networks. Proceedings of the 30th IEEE/CVF Conference on Computer Vision and Pattern Recognition (CVPR).

[48] Liu M., Zhang Z., Hou L., Zuo W., Zhang L. Deep adaptive inference networks for single image super-resolution. Proceedings of the European Conference on Computer Vision.

[49] Bevilacqua M., Roumy A., Guillemot C., Alberi-Morel M.L. Low-complexity single-image super-resolution based on nonnegative neighbor embedding. Proceedings of the 23rd British Machine Vision Conference, University of Surrey.

[50] Jang E., Gu S., Poole B. (2016). Categorical reparameterization with gumbel-softmax. arXiv.

[51] Agustsson E., Timofte R. Ntire 2017 challenge on single image super-resolution: Dataset and study. Proceedings of the IEEE Conference on Computer Vision and Pattern Recognition Workshops.

[52] Zeyde R., Elad M., Protter M. On single image scale-up using sparse-representations. Proceedings of the International Conference on Curves and Surfaces.

[53] Martin D., Fowlkes C., Tal D., Malik J. A database of human segmented natural images and its application to evaluating segmentation algorithms and measuring ecological statistics. Proceedings of the Eighth IEEE International Conference on Computer Vision, ICCV 2001.

[54] Huang J.-B., Singh A., Ahuja N. Single Image Super-Resolution from Transformed Self-Exemplars. Proceedings of the IEEE Conference on Computer Vision and Pattern Recognition.

[55] Fujimoto A., Ogawa T., Yamamoto K., Matsui Y., Yamasaki T., Aizawa K. Manga109 dataset and creation of metadata. Proceedings of the 1st International Workshop on coMics ANalysis, Processing and Understanding.

[56] Kingma D.P., Ba J. (2014). Adam: A method for stochastic optimization. arXiv.

[57] Dong C., Loy C.C., Tang X. Accelerating the Super-Resolution Convolutional Neural Network. Proceedings of the European Conference on Computer Vision ECCV.

[58] Kim J., Lee J.K., Lee K.M. Deeply-Recursive Convolutional Network for Image Super-Resolution. Proceedings of the 2016 IEEE Conference on Computer Vision and Pattern Recognition (CVPR).

[59] Lai W.S., Huang J.B., Ahuja N., Yang M.H. Deep Laplacian Pyramid Networks for Fast and Accurate Super-Resolution. Proceedings of the 30th IEEE/CVF Conference on Computer Vision and Pattern Recognition (CVPR).

[60] Li Z., Yang J.L., Liu Z., Yang X.M., Jeon G., Wu W., Soc I.C. Feedback Network for Image Super-Resolution. Proceedings of the 32nd IEEE/CVF Conference on Computer Vision and Pattern Recognition (CVPR).

[61] Yang Y., Newsam S. Bag-of-Visual-Words and spatial Extensions for Land-Use Classification. Proceedings of the 18th SIGSPATIAL International Conference on Advances in Geographic Information Systems.

[62] Ma Y.C.A., Lv P.Y., Liu H., Sun X.H., Zhong Y.F. (2021). Remote Sensing Image Super-Resolution Based on Dense Channel Attention Network. Remote Sens..

[63] Cheng G., Han J.W., Lu X.Q. (2017). Remote Sensing Image Scene Classification: Benchmark and State of the Art. Proc. IEEE.

[64] Xie C., Zhu H.Y., Fei Y.Q. (2022). Deep coordinate attention network for single image super-resolution. IET Image Process..

[65] Wu Q., Zhang H.R., Zhao W., Zhao X.L. (2020). Shape Optimum Design by Basis Vector Method Considering Partial Shape Dependence. Appl. Sci..

[66] Xiong T.Y., Gu Z. (2020). Observer-Based Fixed-Time Consensus Control for Nonlinear Multi-Agent Systems Subjected to Measurement Noises. IEEE Access.

[67] Zhang Y.Y., Jiang L., Yang W.X., Ma C.B., Yu Q.P. (2020). Investigations of Adhesion under Different Slider-Lube/Disk Contact States at the Head-Disk Interface. Appl. Sci..

